# Osteopathic Manipulative Treatment for Chronic Obstructive Pulmonary Disease: A Systematic Review

**DOI:** 10.7759/cureus.102712

**Published:** 2026-01-31

**Authors:** Alexander Ponce, Ka Hyun Paek, Kevin Heintzelman

**Affiliations:** 1 Internal Medicine, William Carey University College of Osteopathic Medicine, Hattiesburg, USA; 2 Internal Medicine, Baptist Memorial Health Care, Jackson, USA

**Keywords:** copd: chronic obstructive pulmonary disease, osteopathic manipulative treatment (omt), osteopathy treatment, six-minute walk test, "spirometry"

## Abstract

Chronic obstructive pulmonary disease (COPD) is a progressive respiratory condition associated with significant morbidity and functional impairment. Osteopathic manipulative treatment (OMT) has been proposed as an adjunctive therapy to address musculoskeletal and respiratory mechanics in patients suffering from COPD. However, the clinical evidence remains limited. Therefore, the objective of this study is to systematically review available clinical evidence evaluating the utility of osteopathic manipulative treatment in patients with chronic obstructive pulmonary disease. A systematic review of the MEDLINE/PUBMED, Google Scholar, Cochrane, and clinicaltrials.gov databases was conducted to identify clinical trials evaluating osteopathic manipulative treatment in adult patients with COPD. Eligible studies were screened and synthesized narratively due to heterogeneity in study design and outcome measures. Ultimately, five clinical studies met the inclusion criteria. Reported outcomes included pulmonary function measures, functional capacity, and patient-reported outcomes. Findings were mixed, with some studies demonstrating improvements in functional and subjective measures, while other studies showed no reported benefit following OMT. Overall, evidence regarding osteopathic manipulative treatments in chronic obstructive pulmonary disease remains limited and mixed. While some studies suggest potential benefit, most notably a statistically significant improvement in the six-minute walk test (6MWT), other studies show no improvement compared with standard care, and larger high-quality trials are needed to clarify OMT’s role as an adjunctive therapy in COPD management.

## Introduction and background

Chronic obstructive pulmonary disease (COPD) is one of the top three causes of death globally [1]. COPD is caused by small airway obstruction and alveolar gas exchange issues [2]. This airway obstruction is driven by chronic airway inflammation in response to prolonged exposure to harmful particles, such as cigarette smoke. Persistent inflammatory cell infiltration, oxidative stress, and protease-antiprotease imbalance lead to airway wall remodeling, mucus hypersecretion, and destruction of alveolar structures, resulting in reduced elastic recoil and impaired gas exchange [3]. These pathological changes induce progressive airflow limitation characteristic of COPD [1,3]. The airflow can be quantified with spirometry, which measures lung volumes such as forced vital capacity (FVC) and forced expiratory volume in one second (FEV₁), with airflow obstruction defined by a reduced FEV1/FC ratio that indicates impaired expiratory airflow due to a narrowed or collapsed airway [1,4]. Additional assessments of COPD include the six-minute walk test (6MWT), a standardized exercise test used to evaluate functional exercise capacity by measuring the distance walked over six minutes, and the COPD assessment test (CAT), a validated patient-reported questionnaire that captures the impact of COPD on patient quality of life. These assessments serve as adjunctive tools to spirometry by quantifying disease severity and evaluating the functional and symptomatic burden of COPD [5,6].

The obstruction of air flow within the lungs found in COPD is not fully reversible. As a result, there is no known cure for COPD [7]. International guidelines, put out by the Global Initiative for Chronic Obstructive Lung Disease (GOLD), emphasize the need for individualized care for patients [1]. The main goals of care in this patient population are to improve their quality of life, decrease risks of exacerbations, and manage chronic symptoms. In order to achieve this goal, the current standard of care encourages pharmacological care in combination with pulmonary rehabilitation [7].

Current international guidelines support pulmonary rehabilitation as a non-pharmacological intervention for the treatment of chronic obstructive pulmonary disease. Defined by the American Thoracic Society and European Respiratory Society, pulmonary rehabilitation is a holistic approach designed to meet the needs of patients who are suffering from chronic respiratory disease [8]. Rehabilitation includes exercise training to improve patients’ exercise tolerance, which is often limited by reduced lung function, as well as education on behavioral changes that support physical recovery and enhance quality of life [9]. Pulmonary rehabilitation focuses on the idea of improving the patient's overall quality of life as well as their pulmonary function without medication. The effects of COPD are not isolated to the lungs. Many patients with COPD experience accompanying musculoskeletal dysfunctions, ranging from musculoskeletal pain to mechanical restrictions. Obstructive lung disease often leads to reduced chest wall mobility, a well-recognized physiological consequence of chronic airflow obstruction [10]. This restriction is believed to contribute to diminished pulmonary function and may play a role in the overall functional decline seen in COPD patients.

Despite current interventions, there may be additional methods that can further enhance COPD management and improve quality of life. Osteopathic manipulative therapy (OMT) may play a role in the management of COPD. Past clinical studies have evaluated the application of OMT in the treatment and management of COPD [11,12]. OMT is grounded in identifying and treating somatic dysfunction, which is defined as impaired or altered function of the body framework. To address these somatic dysfunctions, Osteopathic physicians utilize a variety of techniques such as soft tissue, myofascial release, high velocity-low amplitude (HVLA), counter strain, lymphatic drainage, and muscle energy [13]. By targeting and restoring normal biomechanical and physiological function, osteopathic medicine may enhance thoracic cage mobility, diaphragmatic excursion, and respiratory muscle flexibility, which are known to be compromised in COPD [11,13]. However, prior studies have reported mild adverse effects and occasional transient worsening of air trapping, emphasizing the importance of careful selection of technique and appropriate patient selection [14]. A 2016 review found that OMT was associated with significant improvements in patient-oriented outcomes, but showed limited effects on disease-oriented measures [15]. Since that review, additional studies have further examined the role of OMT in COPD management. Therefore, this systematic review synthesizes both earlier and more recent evidence to clarify the efficacy of OMT on lung function, exercise capacity, and patient-reported quality of life with COPD by identifying patterns of benefits across objective and subjective outcome measures.

## Review

Materials and methods

Search Strategy

Studies were obtained from the MEDLINE/PubMed, Google Scholar, Cochrane, and ClinicalTrials.gov research databases. This systematic review was conducted and reported in accordance with the Preferred Reporting Items for Systematic Reviews and Meta-Analyses (PRISMA) guidelines. Searches were conducted in January 2026 using combinations of terms related to chronic obstructive pulmonary disease and osteopathic manipulative treatment. The PubMed, Google Scholar, and Cochrane searches used the following MeSH: (“Pulmonary disease, Chronic Obstructive” OR COPD OR "chronic obstructive pulmonary disease”) and (“osteopathic manipulative treatment” OR “osteopathic manual medicine” OR “manipulation, osteopathic” OR osteopathic OR OMT or OMM). The search of ClinicalTrials.gov used “Chronic Obstructive Pulmonary Disease” as the condition/disease and “Osteopathic Manipulative Treatment” in the other terms section.

Eligibility Criteria

Studies were eligible for inclusion if they involved adult patients with chronic obstructive pulmonary disease, evaluated osteopathic manipulative treatment as an intervention, and reported patient-level clinical or physiological outcomes. Only clinical and randomized controlled trials were included. Narrative reviews, systematic reviews, interviews, and studies evaluating non-osteopathic manual therapies were excluded.

Study Selection and Data Extraction

**Figure 1:**
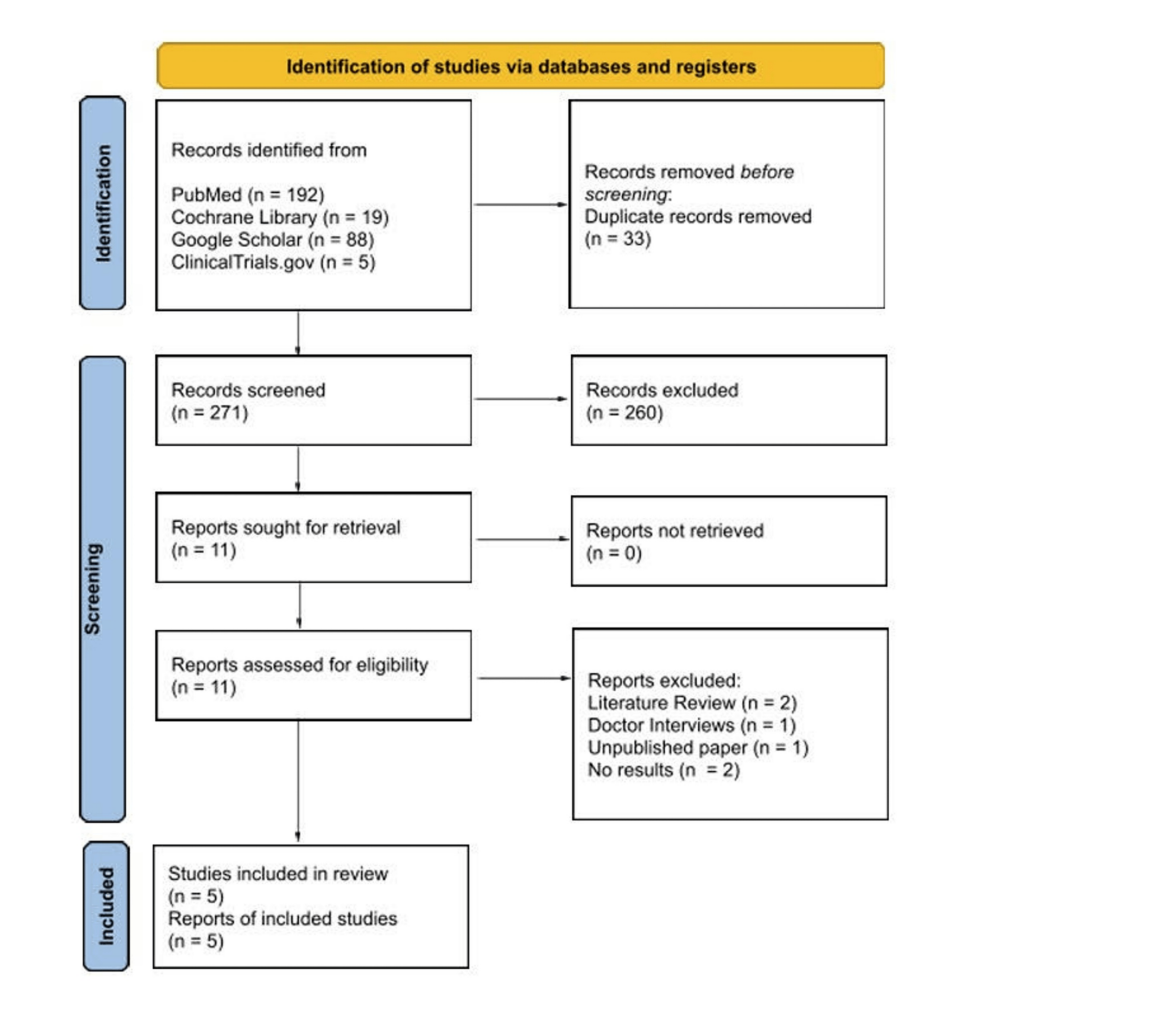
PRISMA flow diagram of study selection.

Literature searches and study selection were performed by two authors (A.P and K.P). Titles and abstracts were screened for relevance, followed by full text review of potentially eligible studies. Studies that did not involve osteopathic manipulative treatment within or did not focus on COPD were excluded. For included studies, data were extracted regarding study design, patient population, sample size, number of OMT sessions, and OMT techniques used. Risk of bias was assessed using the Cochrane Risk of Bias 2 (RoB 2) framework and summarized qualitatively across key domains, including the randomization process, deviations from intended interventions (including blinding), missing outcome data, outcome measurement, and selective reporting. Overall judgments reflected the reported risk of bias within these domains. Due to heterogeneity in study design and outcomes, results were synthesized narratively rather than pooled for meta-analysis. Study selection is summarized in Figure 1.

Results

A total of 304 records were identified through the initial literature search. Thirty-three duplicate records were found and removed. After title and abstract screening, 11 articles were sought for retrieval and underwent full-text review. Five articles were selected based upon the criteria: Two literature reviews, one collection of doctor interviews, one unpublished paper, and two without results were excluded after full text review. While many articles mentioned chronic obstructive pulmonary disease or osteopathic manipulative treatment, few evaluated osteopathic manipulative treatments as an intervention for COPD. Summaries of the five studies, including their respective study design, sample size, patient population, outcomes reported, and key findings, are shown in Table 1 below. Risk of bias was assessed using the RoB 2 tool across five predefined domains, with overall judgments assigned according to Cochrane recommendations. Table 2 outlines the five domains and overall judgments of the included studies.

**Table 1:** Characteristics of included studies

Author	Study Design	Population	Sample Size	Number of OMT Sessions	OMT Techniques Used	Outcomes Measured	Key Findings
Zanotti et al. [16]	Randomized ControlledPilot study	Adult patients with stable COPD varying severities	N = 20	4	Unique OMT for each individual in study	Spirometry (VC, FVC, FEV1 and RV) and 6-Minute Walk Distance	The addition of osteopathic manipulative treatment (OMT) to standard therapy resulted in a significantly greater improvement in 6-minute walk distance compared with standard therapy alone (72.5 ± 7.5 m vs 23.7 ± 9.7 m; p=0.01). Standard therapy alone was not associated with significant changes in spirometric values. In contrast, the standard therapy plus OMT group demonstrated a reduction in residual volume of approximately 11% (p=0.05). Although FEV₁ improved by 14% in the OMT group, the difference compared with standard therapy alone was not statistically significant.
Buscemi et al. [17]	Randomized Controlled Pilot Study	Patients with moderate to severe COPD	N = 32	1	Maxillary bone technique, Vertebral-pleural ligament technique, Phrenic nerve technique, Conoid ligament technique, Trapezoid ligament technique, Subclavius muscle technique, Lung technique, Bronchus technique, Pleura technique, Rib technique	Spirometry (FVC, FEV1), 6-Minute Walk Test/6MWT, and COPD Assessment Test/CAT questionnaire	Spirometry showed improved, but not statistically significant, FVC (p<0.5411) and total FEV1 (p<0.5061). Statistically significant improvement in distance during 6MWT (p<0.0038) in comparison to the control group. CAT questionnaire results showed statistically significant improvement (p<0.05)
Noll et al. [14]	Double-blind, randomized controlled trial	Diagnosis of COPD, 65 years of age, and airflow obstruction present	N = 35	1	Soft tissue to paraspinal muscles, Rib raising, Redoming of the abdominal diaphragm, Suboccipital decompression, Thoracic inlet myofascial release, Pectoral traction, and Thoracic lymphatic pump with activation	Spirometry (FVC, ERV, RV, FEF, and TLC) and telephone survey	FEF 25% and FEF 50% both had statistically significant change (p=0.04) and (p=0.008) respectively. Other lung measurements demonstrated statistically significant increases in RV, TLC, FVC, and ERV. Telephone survey results were not statistically significant.
Maskey-Warzechowska et al. [18]	Randomized crossover trial	Adult patients (mean age of 68) with stable, severe-to-very severe COPD; substantial smoking history	N = 19	1	Suboccipittal decompression, deep cervical fascia release, thoracic lymphatic pump, and Diaphragm “stretching”	Spirometry, body plethysmography, and dyspnea assessment (VAS)	No significant overall improvement in pulmonary function or dyspnea after a single OMT session. 36.8% of the participants achieved a clinically meaningful reduction in RV (>6.1%) following OMT; also exhibited higher baseline dyspnea. However, similar RV reductions after sham therapy suggest nonspecific treatment effects.
Górska et al. [19]	Randomized cross-over trial	Patients with severe COPD	N = 19	1	Not specified	Spirometry, Dyspnea	No significant immediate benefit of OMT on spirometry or dyspnea compared with sham in the overall population.

**Table 2:** Risk of bias assessment of included randomized controlled trials using the Cochrane Risk of Bias 2 (RoB 2) tool.

Study	Randomization process	Deviations from intended interventions	Missing outcome data	Measurement of the outcome	Selection of the reported result	Overall risk of bias
Zanotti et al. [16]	Low risk	Low risk	Low risk	Low risk	Some concerns	Some concerns
Buscemi et al. [17]	Some concerns	High risk	Low risk	Some concerns	Some concerns	High risk
Noll et al. [14]	Low risk	Low risk	Low risk	Low risk	Some concerns	Some concerns
Maskey-Warzechowska et al. [18]	Low risk	Some concerns	Low risk	Low risk	Some concerns	Some concerns
Górska et al. [19]	Some concerns	Some concerns	Some concerns	Low risk	Some concerns	Some concerns

Discussion

This systematic review evaluated the efficacy of OMT as a supplemental intervention for patients with COPD, with a focus on pulmonary function, exercise capacity, and patient-reported outcomes. Across the five included studies, the effects of OMT were variable and appeared to be context-dependent, with outcomes differing based on the type of measurement assessed. Given the methodological heterogeneity of the available studies, including the use of differing objective and subjective outcome measures, each outcome was examined separately. This approach allowed for clear comparison of methods, measured effects, identification of potential sources of variability, and implications for the role of OMT in COPD management. Overall, findings from the five selected studies suggest that OMT may offer selective and context-dependent benefits, though evidence remains limited and methodologically heterogeneous. This is reflected in measured outcomes from spirometry, 6-minute walk tests, and survey/questionnaire-type assessments.

Spirometry

Spirometry served as a primary objective measure of pulmonary function across the five studies. Zanotti et al. reported that adding OMT to pulmonary rehabilitation (PR) resulted in selective improvements, including approximate 14% increase in FEV₁ (0.99 ± 0.4 L to 1.13 ± 0.4 L) and a significant reduction in residual volume (RV) (4.4 ± 1.5 L to 3.9 ± 1.5 L, p=0.05), with a robust difference between the OMT+PR group and PR only group (-0.44 L; p=0.001), suggesting reduced hyperinflation not observed with PR alone [16]. However, changes in FVC were not statistically significantly different between groups. In contrast, Buscemi et al. observed modest changes that did not reach statistical significance for FVC (p<0.54) or FEV₁ (p<0.51) [17]. Furthermore, Noll et al. demonstrated significant immediate changes following OMT that indicated worsened condition, which is shown in the reductions in mid-expiratory flow rates represented by FEF₅₀% (-10.5% vs +6.3%, p=0.02) and FEF₂₅-₇₅% (-12.0% vs +3.2%, p=0.04), alongside significant increases in RV, TLC, and RV/TLC, which indicates worsened air trapping immediately post-treatment [14]. Additionally, Maskey-Warzechowska et al. found no significant changes in FEV₁, FVC, or FEV₁/FVC, and no significant reductions in RV (p=0.23) [18]. Likewise, body plethysmography demonstrated no significant group-level changes in RV or RV/TLC following OMT compared with sham treatment. Although 36.7% of participants exceeded the minimally important difference (-6.1% predicted), comparable changes were shown in a similar portion (47.4%) in the sham group [18]. This suggests individual variability without a clear OMT effect across all patients. Furthermore, Gorska et al. failed to demonstrate significant improvements in pulmonary function following OMT [19]. This suggests that short-term osteopathic treatment may be insufficient to elicit measurable changes and that extended treatment protocols may be necessary. Collectively, spirometric outcomes following OMT were heterogeneous and failed to demonstrate consistent immediate improvements in airflow or lung volumes, suggesting that OMT may not reliably influence fixed obstructive physiology in COPD.

Six-Minute Walk Test

The six-minute walk test (6MWT), a common measure of functional exercise capacity of patients with pulmonary disease, was used by Zanotti et al. and Buscemi et al [16,17]. In Zanotti et al., 6MWT improvement was shown in the PR+OMT group compared to the PR only group [16]. The PR+OMT group improved with an approximate distance of 50m as opposed to 20m performed by the PR only group. The between-group difference, therefore, was statistically significant (p<0.05). Most importantly, this exceeded the common minimal important difference as noted by Holland et al., which was about 25m [20]. Furthermore, Buscemi et al. also demonstrated a statistically significant improvement in distance (p<0.004) in comparison to the control group [17]. Together, these findings suggest that the addition of OMT may provide additional benefit in functional exercise capacity. The reproducible improvement in 6MWT distance highlights functional performance as a meaningful, quantifiable outcome that may reflect therapeutic benefit beyond what is captured by spirometric measurements.

Patient Reported Outcomes

Another measure of pulmonary function, specifically its impact on the participants’ quality of life, was assessed via surveys. In Noll et al., aside from two participants complaining of muscle soreness, the overall post-treatment survey data indicated perceived benefit following OMT, despite unfavorable spirometry measurements as mentioned earlier [14]. 82% of the OMT group reported better breathing after treatment, compared to the 65% of the sham group that reported the same. Moreover, Buscemi et al. also utilized a survey, specifically the COPD Assessment Test, which consisted of questionnaires, and the results showed statistically significant improvement [17]. Lastly, Maskey-Warzechowska et al. assessed dyspnea using the visual analogue scale (VAS), which did not demonstrate significant differences between OMT and sham assignments [18]. Similarly, Gorska et al. also assessed dyspnea and did not find a difference between OMT and sham treatments [19]. Differences between subjective patient-reported improvements and objective physiologic measures, including spirometry and the six-minute walk test, may reflect discrepancies in patient expectations and response-related effects. Under these circumstances, patients may perceive symptomatic improvement in the absence of measurable changes in spirometric values, walking distance, or chest wall mechanics. This pattern may be influenced by placebo effects or reporting bias, both of which are common considerations when interpreting survey-based outcomes.

Limitations

Although these five studies suggest that OMT may have variable effects in patients with COPD, each study is subject to notable methodological limitations. All studies had small sample sizes (ranging from 19 to 35), impacting the statistical power and generalizability. Our search included PubMed/MEDLINE, the Cochrane Library, Google Scholar, and ClinicalTrials.gov. We did not search additional subscription-based databases, and some relevant studies may have been missed. Additionally, patient-reported outcomes, while clinically relevant, are inherently subjective and rely on individual patient perception without a standardized physiologic correlation. As a result, these measures may be influenced by expectation effects, placebo response, and reporting bias, which can influence interpretation when objective improvements are not concurrently observed. In addition, the number of OMT sessions provided to the participants varied among studies. The only study that offered more than one session was Zanotti et al., which offered four sessions [16]. This was higher than a single OMT session offered by the other studies [14,17-19]. Other differences between studies include variations in specific OMT techniques used, with some studies, like Noll et al., utilizing a standardized OMT protocol for every patient, while others, including Zanotti et al., tailored OMT to each patient [14,16]. The combination of the small sample size and the limited OMT exposure limits the impact and generalizability of the findings. However, despite these limitations, OMT was well tolerated among participants across studies, with no significant adverse events reported.

## Conclusions

Current evidence evaluating the role of osteopathic manipulative treatments in COPD remains limited due to the few studies and inconsistent results. While available data suggest that OMT may be associated with improvements in select outcomes, the clinical significance of these effects remains uncertain. Notably, patients receiving OMT had statistically significant improvements in their 6MWT. Larger, high-quality, adequately powered trials are needed to better define the role of osteopathic manipulative treatments as an adjunctive therapy in COPD management.
